# Altered Spontaneous Brain Activity in Patients with Parkinson’s Disease Accompanied by Depressive Symptoms, as Revealed by Regional Homogeneity and Functional Connectivity in the Prefrontal-Limbic System

**DOI:** 10.1371/journal.pone.0084705

**Published:** 2014-01-03

**Authors:** Ke Sheng, Weidong Fang, Meilan Su, Rong Li, Dezhi Zou, Yu Han, Xuefeng Wang, Oumei Cheng

**Affiliations:** 1 Department of Neurology, The First Affiliated Hospital, Chongqing Medical University, Chongqing, China; 2 Department of Radiology, The First Affiliated Hospital, Chongqing Medical University, Chongqing, China; University of Toronto, Canada

## Abstract

As patients with Parkinson’s disease (PD) are at high risk for comorbid depression, it is hypothesized that these two diseases are sharing common pathogenic pathways. Using regional homogeneity (ReHo) and functional connectivity approaches, we characterized human regional brain activity at resting state to examine specific brain networks in patients with PD and those with PD and depression (PDD). This study comprised 41 PD human patients and 25 normal human subjects. The patients completed the Hamilton Depression Rating Scale and were further divided into two groups: patients with depressive symptoms and non-depressed PD patients (nD-PD). Compared with the non-depressed patients, those with depressive symptoms exhibited significantly increased regional activity in the left middle frontal gyrus and right inferior frontal gyrus, and decreased ReHo in the left amygdala and bilateral lingual gyrus. Brain network connectivity analysis revealed decreased functional connectivity within the prefrontal-limbic system and increased functional connectivity in the prefrontal cortex and lingual gyrus in PDD compared with the nD-PD group. In summary, the findings showed regional brain activity alterations and disruption of the mood regulation network in PDD patients. The pathogenesis of PDD may be attributed to abnormal neural activity in multiple brain regions.

## Introduction

Up to 45% of Parkinson’s disease (PD) patients develop depression [1], but the etiology for this is unclear [2]. The onset of depression occurs early, prior to the onset of motor symptoms [3]. PD with depression (PDD) may represent a specific subgroup of PD patients [4]. It is unclear whether PD and depression have common pathophysiological pathways. Functional neuroimaging approaches have been applied to study in PD patients with depression [5], [6]. The Positron-Emission Tomography (PET) studies have highlighted the involvement of serotonergic systems in PDD in the median raphe nuclei and limbic structures, which is similar to depression in non-PD patients [7], [8]. A volumetric magnetic resonance imaging (MRI) study suggested that there is a negative correlation between the depression severity and gray matter density in the right rectal gyrus and bilateral middle/inferior orbitofrontal regions in PDD [9]. In a recent voxel-based morphometry study, Kostic et al. found that loss of white matter within the cortical–limbic network was positively associated with PDD [10]. A event-related fMRI study found that there are changed activities in the left mediodorsal thalamus and in medial prefrontal cortex in PDD compared with those without depression [6]. A recent study showed that depressed PD patients had significantly decreased amplitude of low-frequency fluctuations in the dorsolateral prefrontal cortex, ventromedial prefrontal cortex and rostral anterior cingulated cortex compared with nD-PD patients [5]. These neuroimaging studies indicated that the prefrontal- limbic system contributes to mood network dysregulation in PDD patients.

Resting-state functional MRI allows investigation of large-scale functional networks at the whole brain level based on the temporal correlation of spontaneous, blood oxygen level-dependent (BOLD) fluctuations in low frequencies (<0.08 Hz) [11], [12], [13]. Resting-state functional MRI (R-fMRI) reflects spontaneous neuronal activity [14], and/or the endogenous or background neurophysiological processes of the brain [11], [15]. Functional impairment has been observed in fMRI studies on PD [16], [17], [18]. Previous R-fMRI studies focused on motor symptoms, but little attention has been paid to depression in PDD.

Regional homogeneity (ReHo) is based on data-driven approach and thus requires no prior knowledge and have good test-retest reliability [19], thus, it is more suitable for the study of a disease with unclear pathological mechanism such as PDD. ReHo [20] is suggested to evaluate the similarity between the time series of a given voxel and its nearest neighbors [21] and reflect the temporal homogeneity of the regional BOLD signal. Changed ReHo value implies changed hemodynamic response. ReHo supposed that voxels within a functional brain area were more temporally homogeneous when this area is involved in a specific condition [20]. This method has been used to explore the functional regulation and to characterize the pathophysiological changes in the resting state in patients with: Alzheimer's disease [22], PD [17], [23], autism spectrum disorders [24], [25] and attention-deficit/hyperactivity disorder [26].

The present study used R-fMRI to examine human regional homogeneity and functional connectivity in non-depressed PD (nD-PD) patients, PDD patients and normal control (NC) subjects. We hypothesized that: PDD patients would show ReHo differences in prefrontal-limbic systems; and connectivity analysis in the PDD group would reveal mood regulation network disruption.

## Materials and Methods

### Participants

This study comprised 41 human patients with idiopathic PD (26 males, 15 females, mean age of 56.6 years, age range 41–65 years). A diagnosis of PD was made based on: medical history; physical and neurological examinations; response to levodopa or dopaminergic drugs; and findings of laboratory tests and MRI scans conducted to exclude other diseases. All patients fulfilled the UK Parkinson’s Disease Society Brain Bank criteria for idiopathic PD [27]. Patients were excluded if they had used antidepressants in the year preceding the study, or if they had cerebrovascular disorders, a history of traumatic brain injury, dementia, seizures, or other neurological or medical disease. In addition, to reduce the influence of aged related cognitive and cerebrovascular degeneration or motion artifacts during MRI scan, patients older than 65 years and patients with severe motor symptoms were excluded. So additional inclusion criteria were as follows: (1) age range from 40 to 65 years; (2) a H&Y stage equal to or less than 3.0 while in an “off” state; and (3) disease duration of less than 10 years. Patients were divided into two groups: those with depression (PDD group) and those without (nD-PD group). A diagnosis of depression was made using the Diagnostic and Statistical Manual of Mental Disorders version four (DSM-IV) criteria [28]. Shortly, all PDD patients must have one or more of the two core criteria (depressed mood, loss of interest or pleasure) and last for more than two weeks. Neurological evaluation, which was conducted during the “off” medication state (wherein subjects refrained from taking their PD medications for at least 12 hours prior to assess), included the Hoehn and Yahr (H&Y) scale [29] and the unified Parkinson’s disease rating scale (UPDRS III) and the Mini-Mental State Examination (MMSE) [30]. The patients then were administered the Hamilton Depression Rating Scale (HAMD) [31] and the self-rating depression scale (SDS) [32]to assess their depression. All neuropsychological evaluation and fMRI scans (for ReHo and functional connectivity analysis) were implemented around the same time.

25 normal subjects (16 males, 9 females, mean age of 56.7 years, age range 49–65 years) who were matched in terms of age and sex with patients served as controls. All normal subjects had a normal neurological status and were without history of neurological or psychiatric diseases. Detailed neuropsychological examination included MMSE, HAMD and SDS were used to excluded dementia and depression in all normal subjects.

All subjects recruited into the study gave their written informed consent approved by the Ethics Committee of the First Affiliated Hospital, Chongqing Medical University in China in accordance with the Declaration of Helsinki.

### Data Acquisition

All magnetic resonance images were acquired using a GE Signa HDxt 3.0 T scanner (General Electric Medical Systems, USA) with a standard eight-channel head coil. Foam padding was used to minimize head motion and noise. High-resolution 3D-T1 (repetition time [TR] = 8.3 ms, echo time [TE] = 3.3 ms, flip angle = 15° thickness/gap = 1.0/0 mm, field of view [FOV] = 240×240 mm, matrix = 256×192) and conventional MRI (T2-weighted FLAIR, TR = 8000 ms, TE = 126 ms, TI = 1500 ms, thickness/gap = 5.0/1.5 mm, FOV = 240×240 mm, matrix = 256×192) images were acquired. R-fMRI data were acquired using an echo-planar image (EPI) pulse sequence with the following parameters: 33 axial slices, thickness/gap = 4.0/0 mm, in-plane resolution = 64×64 pixels, TR = 2000 ms, TE = 40 ms, flip angle = 90°, FOV = 240×240 mm. A total of 240 time points were obtained (duration = 8 min). During R-fMRI acquisition, all subjects were asked to relax, remain still with their eyes closed, and not to move or fall asleep.

### Data Processing

The data were analyzed using Statistical Parametric Mapping (SPM8) (http://www.fil.ion.ucl.ac.uk), Resting state fMRI data analysis Toolkit (REST) software [33] (http://www.restfmri.net), and the Data Processing Assistant for Resting-State fMRI - Advanced (DPARSFA; http://www.restfmri.net), [34] with Matlab version 7.10.0.499 [35].

The first 10 time points were discarded to account for scanner calibration and the acclimatization of subjects to the scanning environment, after which 230 time points remained. The preprocessing procedures included: time alignment across slices, motion correction, within-subject registration between T1 and EPI images, T1 segmentation, and the application of normalization parameters to the BOLD fMRI datasets to register them to Montreal Neurologic Institute (MNI) space, with voxels resampled at 3×3×3 mm. Linear trends were removed and a temporal filter (0.01 Hz<*f*<0.08 Hz) was applied to eliminate low frequency drift and physiological high frequency noise. Head motion can influence on result even though traditional realignment was performed [36], [37]. All images were realigned to the first image to account for head motion. All subject had a maximum displacement in any of the cardinal directions (x, y, z) less than 2 mm, or a maximum spin (x, y, z) less than 2°. In addition, following previous studies [38], the mean relative displacement was used to measure subjects’ head motion in scanner.

### ReHo Analysis

Individual ReHo maps were generated for each subject using the REST software; Kendall’s coefficient of concordance (KCC) was calculated at each voxel to establish similarities between the time series of each specific voxel and its 26 neighboring voxels [20]. The KCC value was calculated to this voxel, and an individual KCC map was obtained for each subject. To reduce the influence of individual variations in the KCC value, ReHo maps normalization was performed by dividing the KCC among each voxel by the averaged KCC of the whole brain. The calibrated ReHo maps were further smoothed using an isotropic Gaussian kernel with a full-width at half maximum (FWNM) of 4×4×4 mm [25].

### Functional-connectivity Analysis

Five regions with PDD group vs nD-PD group ReHo significantly differences were defined as regions of interest (ROIs). Five areas were selected as seed regions based on the ReHo findings. These included the left middle frontal gyrus, right inferior frontal gyrus, left amygdala and bilateral lingual gyrus. The ROIs were used as the seeds for functional connectivity analysis in the resting state using REST. A seed reference time course was obtained by averaging the time courses within each ROI. Correlation analysis was carried out between the seed reference and the whole brain in a voxel-wise manner [39]. In the correlation analysis, eight nuisance covariates were regressed, including: the white matter signal, the cerebrospinal fluid signal, and six head motion parameters.

### Statistical Analysis

Differences of age, MMSE scores, HAMD scores and SDS scores among the three groups were compared by using one-way analysis of variance (ANOVA), and Pearson *x*2 test was applied to compare patient medications. Student t test was employed to compare the illness duration and disease stage between PDD and nD-PD group.

An ANOVA was performed on the resting-state REST data to identify the ReHo maps from the patients and control group. Voxels with a p value less than 0.05 and a cluster size greater than 1836 mm^3^ (68 voxels) were considered significantly different, corresponding to a corrected p value less than 0.05 as determined by AlphaSim correction in REST software. Subsequently, the regions that showed significant differences were extracted as a mask, and the RS-fMRI measures (i.e., ReHo values and the strength of the functional connectivity) were subjected to post hoc analysis. Statistical comparisons of the RS-fMRI measures between each pair of groups (PDD vs nD-PD, PDD vs NC, nD-PD vs NC) were performed using a two-sample post hoc t-test. Corresponding to a corrected p value less than 0.05 as determined by AlphaSim correction, a cluster size greater than 1755 mm^3^ (65 voxels) were considered significantly different.

## Results

### Demographic and Clinical Data

We acquired resting fMRI data from 41 patients and 25 normal controls. Twenty of the patients were classified as depressed according to the DSM-IV criteria. PDD showed higher HAMD and SDS scores compared with those with nD-PD group (P<0.001). According to the DSM-IV criteria, all patients in PDD group had a minor depressive disorder. There was no difference between the depressed and non-depressed patient groups in terms of: duration of PD, H&Y stage, side initially affected, medication or MMSE score (P>0.05). Importantly, depressed PD patients were matched regarding PD motor severity (according to UPDRS III) to nD-PD patients. Age and gender did not differ in PD groups with controls as well as between patient groups (Table 1).

**Table 1:** Demographic information for the PDD/nD-PD and control groups.

Group N	PDD	nD-PD	NC	P-Value
(Male/Female)	N = 20 (13 m/8 f)	N = 21 (13 m/7 f )	N = 25 (16 m/9 f )	
Age (Y)	55.9±7.4	57.3±6.1	56.7±5.3	0.250 ★
Disease duration (Y)	3.4±1.7	4.0±2.4	NA	0.224 ▴
Disease stage (H&Y)	2.1±0.75	1.95±0.63	NA	0.154 ▴
Side initially affected, R/L	12/8	11/10	NA	0.623 •
UPDRS III	39.4±10.8	43.8±8.2	NA	0.078 ▴
MMSE (Mean ±Sem)	26.9±1.7	27.6±2.0	29.2±0.9	0.096 ★
HAMD (Mean ± Sem)	19.3±5.0	6.4±2.1	5.6±1.9	<0.001 ★
SDS (Mean ± Sem)	64±5.5	29.6±5.3	25.3±0.9	<0.001 ★
L-Dopa dose (mg/d)	406.2±171.5	398.8±242.2	NA	0.315 ▴
No. (%) of patients treated with pramipexole	17(85)	15(75)	NA	0.421 •
No. (%) of patients treated with piribedil	7(35)	6(30)	NA	0.700 •

NC: normal control, PDD: Parkinson disease patients with depression, nD-PD: non-depressed Parkinson’s disease patients, NA: not applicable, ★: one-way analysis of variance (ANOVA), ▴: two sample t-test, •: Pearson *x*2 test.

### Head Motion

There was no significant difference in head motion by measured mean head motion between the three groups using ANOVA analysis (P = 0.31). Therefore, the patients with PD and normal controls in the present study were similar in head motion characteristics.

### ReHo

**Figure 1:**
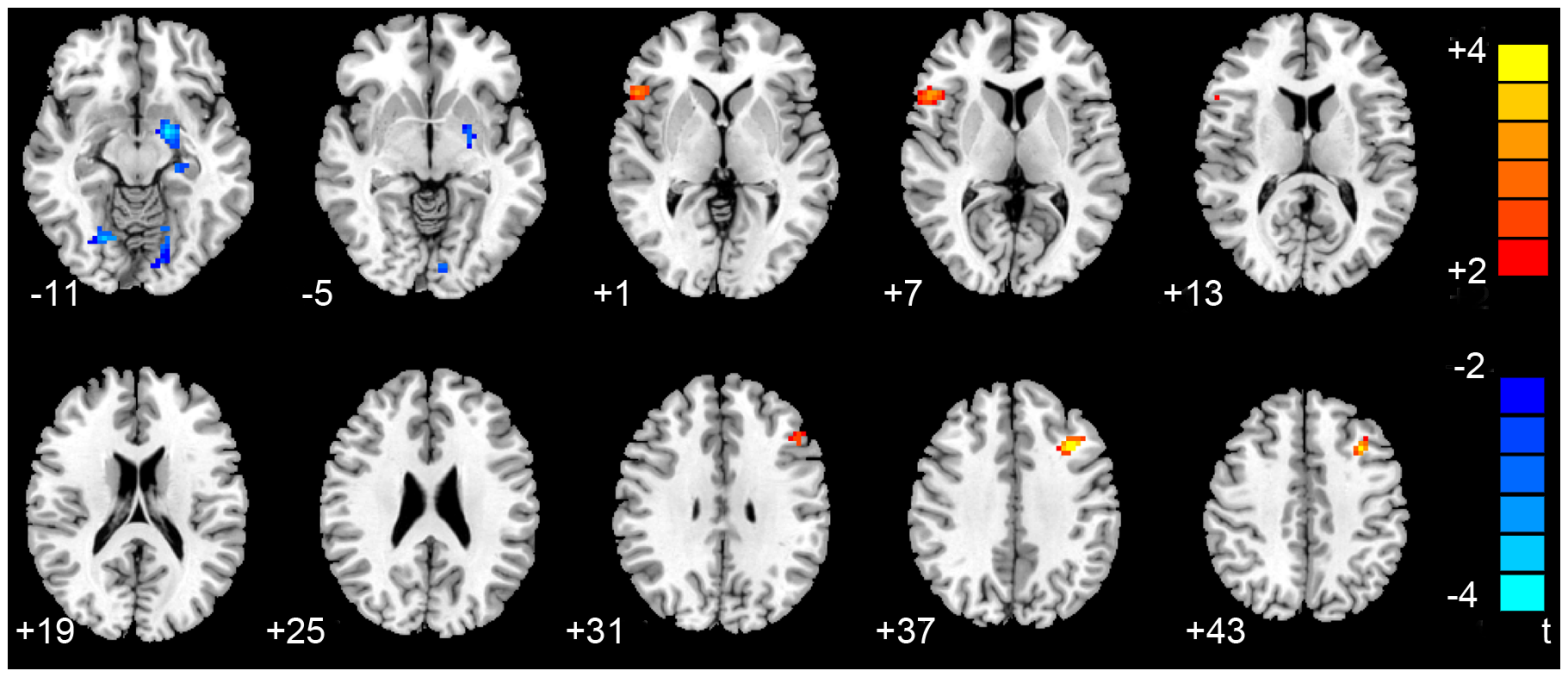
Differences in ReHo values between the PDD and nD-PD groups. (P<0.05, AlphaSim corrected).

An ANOVA revealed significant differences in the ReHo index between the PDD, nD-PD and NC groups in the following regions: bilateral pallidum, bilateral insula, bilateral precentral gyrus, bilateral lingual gyrus, bilateral cerebellum, left amygdala, left middle frontal gyrus, left hippocampus, right inferior frontal gyrus and right superior frontal gyrus (P<0.05; AlphaSim corrected). A two-sample two-tailed t-test was then performed to determine differences in the fitted mean ReHo indices for each pair of (PDD, nD-PD and NC) groups. Compared with the nD-PD group, the PDD group showed increased regional activity in the left middle frontal gyrus and right inferior frontal gyrus, and decreased ReHo values in the left amygdala and bilateral lingual gyrus (P<0.05, AlphaSim corrected; Table 2 and Figure 1). Compared with the NC group, the PDD patients showed significant ReHo increases in the right cerebellum and right inferior frontal gyrus, and decreases in the bilateral pallidum, bilateral precentral gyrus, left hippocampus, left insula and right lingual gyrus (AlphaSim corrected, P<0.05; Table 2). In addition, compared with the NC group, the nD-PD group’s ReHo values were significantly increased in the right superior frontal gyrus and bilateral cerebellum, and no region decreased (AlphaSim corrected, P<0.05; Table 2). The details of the peak coordinates and cluster sizes are listed in Table 2.

**Table 2:** Brain regions exhibiting decreased and increased regional homogeneity among three groups.

Brain region	Brodmann area		Cluster size		MNI			T value
					x	y	z	
**PDD<nD-PD**								
Left lingual gyrus		18	95	−18		−79	−13	−3.98
Left amygdala		34	105	−20		−4	−15	−3.81
Right lingual gyrus		18	89	13		−87	−7	−3.44
**PDD>nD-PD**								
Left middle frontal gyrus		46	98	−36		19	39	4.44
Right inferior frontal gyrus		45	101	49		25	5	3.52
**PDD<NC**								
Left hippocampus		20	107	−32		−20	−13	−5.01
Left pallidum			103	−23		−1	0	−4.26
Left insula		48	108	−37		−14	12	−3.87
Right precental gyrus		6	111	51		5	45	−3.81
Right pallidum			111	19		6	0	−3.63
Right fusiform gyrus		37	97	26		−63	−11	−3.61
Left precental gyrus		6	107	−48		−5	46	−3.54
**PDD>NC**								
Right inferior frontal gyrus		45	98	53		21	2	4.20
Right cerebellum			109	37		−50	−45	3.09
**nD-PD >NC**								
left cerebellum			90	−19		−50	−55	3.66
Right cerebellum			91	20		−49	−55	3.53
Right superior frontal gyrus		8	115	10		27	54	3.38

NC: normal controls, PDD: Parkinson’s disease patients with depression, nD-PD: non-depressed Parkinson’s disease patients. A>B: Compared with B group, A group showed increased ReHo values. A<B: Compared with B group, A group showed decreased ReHo values (P<0.05, AlphaSim corrected).

### Functional Connectivity

**Figure 2:**
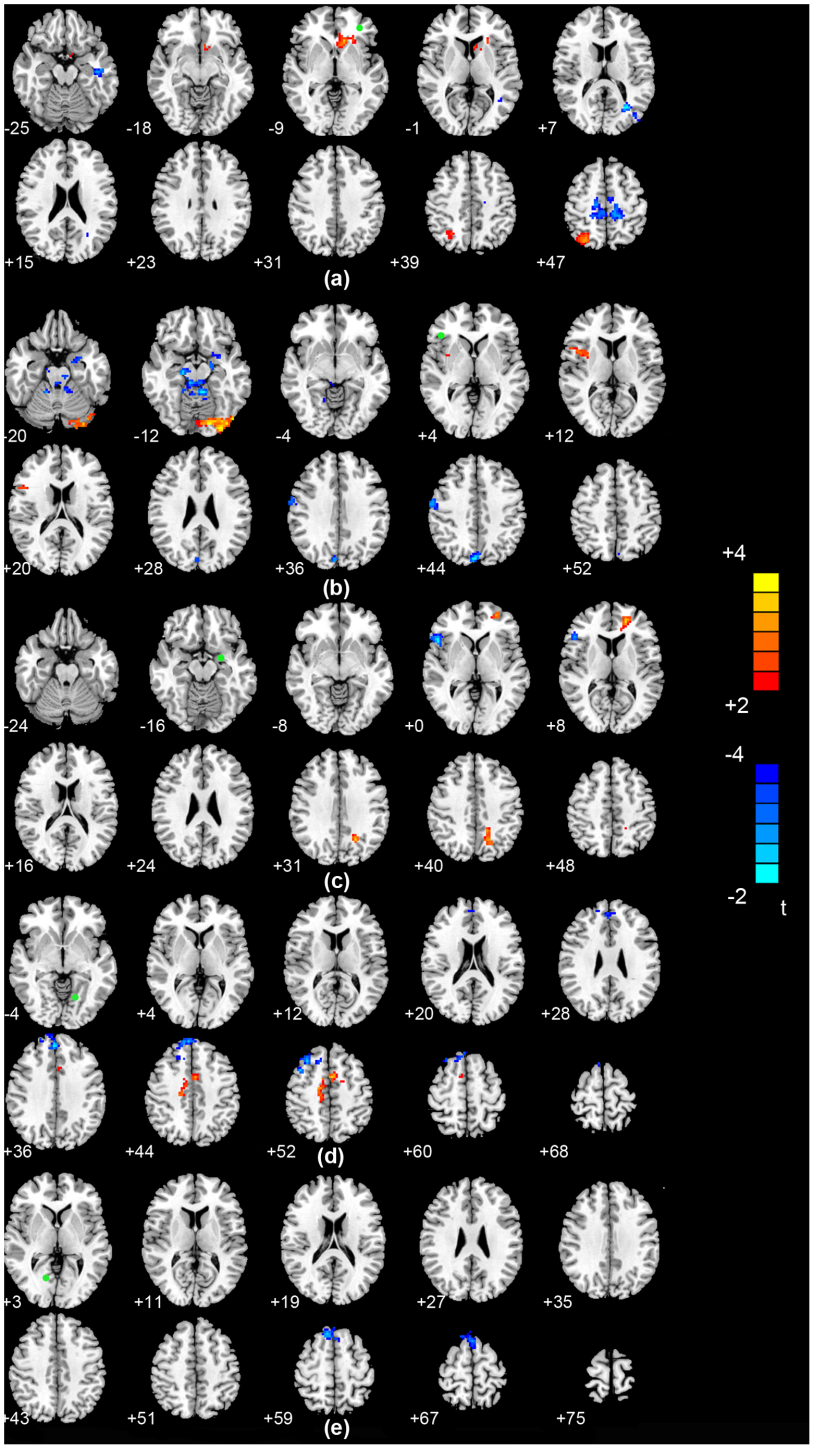
Statistical parametric map showing the significant differences of functional connectivity between PDD and nD-PD groups. (a) Differences in functional connectivity for the left middle frontal gyrus seeds between the PDD and nD-PD groups. (b) Differences in functional connectivity for the right inferior frontal gyrus between the PDD and nD-PD groups in the resting state. (c) Differences in functional connectivity for the left amygdala seeds between the PDD and nD-PD groups. (d) Differences in functional connectivity for left lingual gyrus seeds between the PDD and nD-PD groups. (e) Differences in functional connectivity for right lingual gyrus seeds between the PDD and nD-PD groups. T score bars are shown on the right. Green spot: the position of the region of interest.

Functional connectivity analysis in a voxel-wise manner was performed to explore differences in the brain networks of the three groups. Based on the ReHo findings, we defined five regions of interest (ROIs): left middle frontal gyrus, right inferior frontal gyrus, left amygdala and bilateral lingual gyrus. In the PDD group, the left middle frontal gyrus showed significant increased connectivity with the right superior parietal gyrus and left caudate,and significant decreased connectivity with the left inferior temporal gyrus compared with the nD-PD group (P<0.05, AlphaSim corrected; Table 3 and Figure 2a). In the PDD group,the right inferior frontal gyrus showed significant increased connectivity with the left lingual gyrus and right insula, and significant decreased connectivity with the left amygdala, left cerebellum, right cuneus and right precentral gyrus compared with the nD-PD group (P<0.05, AlphaSim corrected; Table 3 and Figure 2b). In the PDD group,the left amygdala showed significant increased connectivity with the left middle frontal gyrus and left superior occipital gyrus, and significant decreased connectivity with the right inferior frontal gyrus compared with the nD-PD group (P<0.05, AlphaSim corrected; Table 3 and Figure 2c). In patients with PDD, the left lingual gyrus showed significant increased connectivity with the bilateral median cingulated gyrus, and significant decreased connectivity with the right superior frontal gyrus and left middle frontal gyrus compared with the nD-PD group (P<0.05, AlphaSim corrected, Table 3 and Figure 2d). Meanwhile, when PDD comparing with nD-PD group, the right lingual gyrus of PDD group has been observed significant decreased connectivity with the right superior frontal gyrus (P<0.05, AlphaSim corrected, Table 3 and Figure 2e). No increased FC was found in the PDD group relative to the nD-PD group (P<0.05, AlphaSim corrected). In addition, for the PDD group, the prefrontal gyrus showed significant increased connectivity with the left hippocampus and lingual gyrus, and had significant decreased connectivity with the left amygdala and left temporal pole compared with the NC group. In the nD-PD group, decreased functional connectivity with the prefrontal gyrus was observed for the basal ganglia and cerebellum, and there was increased functional connectivity with the hippocampus and medial cingulate gyrus compared with the NC group (P<0.05, AlphaSim corrected).

**Table 3:** Differences of functional connectivity between PDD and nD-PD.

Seed	Region	Brodmann area	MNI			Cluster size	T value
			x	y	z		
**Left middle frontal gyrus**							
	Right superior parietal gyrus	7	28	−70	56	118	4.48
	Left caudate	25	−6	22	0	118	4.09
	Left inferior temporal gyrus	20	−46	−15	−20	119	−3.69
**Right inferior frontal gyrus**							
	Left lingual gyrus	18	−32	−93	−14	112	4.18
	Right insula	48	40	8	7	115	3.42
	Right cuneus	7	4	−77	42	93	−4.29
	Right precental gyrus	4	54	−12	44	96	−3.42
	Left amygdala	34	−19	−4	−14	89	−3.17
	Left cerebellum		−27	−77	−30	107	−3.04
**Left amygdala**							
	Left middle frontal gyrus	11	−26	52	5	118	3.69
	Left superior occipital gyrus	7	−18	−63	40	112	3.53
	Right inferior frontal gyrus	45	50	20	0	96	−4.32
**Left lingual gyrus**							
	Right superior frontal gyrus	8	8	27	64	92	−4.61
	Left middle frontal gyrus	8	31	27	52	86	−4.95
	Right median cingulate gyrus		13	−15	52	112	4.87
	Left median cingulate gyrus	24	−5	3	42	84	4.72
**Right lingual gyrus**							
	Right superior frontal gyrus	8	8	27	64	92	−4.61

## Discussion

The present study demonstrated that the ReHo of spontaneous activity in the brain and the patterns of connectivity of brain networks were abnormal in PDD patients during resting state. Compared with the nD-PD group, the PDD patients showed significant ReHo value decreases in the bilateral lingual gyrus and left amygdala, and increases in the left middle frontal gyrus and right inferior frontal gyrus. Functional connectivity analysis revealed decreased connectivity with prefrontal gyrus was observed in PDD in the left amygdala, Left inferior temporal gyrus and left cuneus, while increased connectivity with the prefrontal gyrus was in the right superior parietal gyrus, left lingual gyrus and right insula compared with the nD-PD patients.

The ReHo approach adopted is based on the hypothesis that brain activity occurs in voxel clusters rather than a single voxel, thus the KCC was used to evaluate the similarity of the time series of a given voxel to those of its nearest neighbors [20]. It can reflect neural synchronization of local brain areas. Synchronized oscillatory activity in the cerebral cortex is thought to be essential for coordination and integration across space and time of activity in anatomically distributed but functionally related neural elements [40]. The increased ReHo may reveal abnormal enhancement in the intraregional neural activity. On the contrary, reduced ReHo of the local brain regions reflects the consistency of reduced neuronal activity in those areas which suggests that the brain dysfunction may exist.

In the current study, the ReHo values for the PDD patients were decreased in the left amygdala and bilateral lingual gyrus, and increased in the left middle frontal gyrus and right inferior frontal gyrus. The middle frontal gyrus, inferior frontal gyrus and amygdala have been recognized as the key regions associated with mood regulation [41], [42], [43]. In non-PD patients with a major depressive disorder, the medial and inferior frontal cortex regions appear to be relatively consistently overactive at rest, and overactive during the induction of negative affect [44]. The aberrant ReHo in those regions in our study may represent spontaneous neural dysfunction in mood processing and top-down modulation in PDD. The abnormal spontaneous neural activity in the medial frontal gyrus might be an important factor for the development of depressive symptoms in PD [6], [45]. The results of a diffusion tensor imaging study showed a decrease in fractional anisotropy values in the white matter of the frontal lobes bilaterally in a PDD patient. Fibers to the striatum and thalamus constitute part of the limbic basal ganglia-thalamocortical circuits, which are important for mood regulation [6], [46]. The decreased ReHo in amygdala in the present study reflects the local destruction of the synchronization of spontaneous low-frequency BOLD fluctuations in the region and implies functional deficits. The degeneration of dopaminergic pathways induces an abnormal function of amygdale in PD [47]. The amygdala mediates fear and anxiety processing [48], and functional abnormalities in this region correlate with the severity of endogenous depression [49]. A depressed PD cohort were shown to have lower [11C] RTI-32 binding in the amygdala compared with a non-depressed PD group [50]. Together, the findings suggest that abnormal spontaneous neural activity in the frontal gyrus and amygdala might partly contribute to the pathogenesis of emotional symptoms seen in PD patients.

Compared with the nD-PD group, the PDD patients in the current study showed significant ReHo value decreases in the bilateral lingual gyrus. The lingual gyrus is assumed to play a critical role in the visual recognition circuit [51], [52] and be involved in the perception of mood [53]. The disturbances of the visual recognition network may partly contribute to the dysfunctional emotional behavior of PD patients with depressive symptoms. We therefore speculate that there might be an association between the ReHo value changes in these regions in PDD patients and the mood symptoms seen in these patients. The results support the hypothesis that PD patients with depressive symptoms show disruption of the mood regulation network.

Resting-state functional connectivity refers to temporal correlations between remote brain regions. Using a functional connectivity analysis, a previous study found highly synchronous low frequency fluctuations of resting-state BOLD signals among different cortices in healthy adults [14], [54], [55]. Brain regions with similar functions and known anatomical connections have shown strong correlations at rest [11], [56]. Studies of the interactions between brain areas may provide more valuable information regarding our understanding of functional changes than simply investigating regional brain activity. The current study therefore used a seed-based correlation analysis to explore resting-state functional connectivity patterns of the abnormal ReHo brain regions in PDD and nD-PD patients. On the basis of the ReHo finding, we further explore the alterative connection pathways with other brain regions at the whole brain level using functional connectivity analysis.

Using a functional connectivity analysis in a voxel-wise manner, our study showed altered connectivity between the amygdala and prefrontal gyrus in the PDD group. A previous study of functional connectivity using structural equation modeling indicated a mood processing bias, with disconnection between the amygdala and prefrontal cortex in depression without PD [57]. In addition, Perlman et al. stated that distinguishable patterns of abnormal amygdala–prefrontal cortex circuitry may result in abnormal mood processing and regulation, which may underlie changes from remission to depression in bipolar disorder [58]. Consistent with these studies, alterations in the strength of connectivity between the amygdala and prefrontal brain regions in the current study provide a neural basis for disrupted emotional recognition processing in the PDD patient.

Our findings showed significantly increased connectivity between the right inferior frontal gyrus and the right insula in PDD compared with nD-PD group. Fitzgerald et al. have shown the inferior frontal gyrus to be influenced by both basal activity and by responses to affective stimuli in depressed patients without PD [44]. The insular cortex has also been implicated in the mood regulation network [59], [60], and potentially plays a role in integrating subcortical and cortical mood processes [61]. Suslow et al. have revealed activation in the insular cortex associated with negative priming [62]. Meanwhile, the PDD patient showed decreased FC between the right inferior frontal gyrus and left cerebellum, compared with the nD-PD group in our study. The cerebellum has been demonstrated to be involved in emotion and cognition in recent years [63], [64]. Decreased FC also was observed between the cerebellum and inferior frontal gyrus in treatment-resistant depression patients relative to healthy controls [65]. Hence we speculate that, in our study, altered FC between the right inferior frontal gyrus and left cerebellum may indicate mood dysregulation seen in PDD.

When comparing the PDD group with nD-PD group, a significant increased in functional connectivity between the left middle frontal gyrus and the right parietal gyrus. A previous study emphasized the general role of the right parietal lobe in the regulation of anticipation of negative stimuli [66]. In addition, the parietal lobe appears to be important in attention [67], [68]. Therefore, the impairment of brain regions implicated in the mood regulation network within a PDD patient may be reflected by increased attention towards negative stimuli and decreased responses to positive stimuli.

Our results for the PDD group showed increased functional connectivity with the right inferior frontal gyrus ROIs in the left lingual gyrus, and decreased functional connectivity in the right cuneus compared with nD-PD patients. The lingual gyrus and cuneus are regarded by some as key regions of the visual recognition circuit [69]. The abnormality connectivity of the visual cortical areas including the lingual cortex has been observed in major depression patient without PD [70], which may be related to impaired selective attention and working memory [71]. Cant et al. suggested that the cuneus may be involved in the extraction of object color relatively early in visual processing, whereas information about texture may implicate the lingual gyrus [72]. It is acknowledged that emotional modulation can influence the processing of a visual cue [73]. Our findings may therefore reflect a visual recognition processing characteristic of PD patients with depressive symptoms.

When comparing the PDD group with the nD-PD group, the bilateral lingual cortex showed significant increased connectivity with the bilateral median cingulate gyrus, and significant decreased connectivity with the right superior frontal gyrus and left middle frontal gyrus compared with the nD-PD group. The lingual gyrus were regarded as the key regions related to visual recognition circuit [52] and may be involved in the perception of mood [53]. Previous task related fMRI study noted that an alteration of visual cotex activity in depression without PD was associated with a reduction of distractors processing compared with healthy controls [71]. The middle frontal gyrus and superior frontal gyrus assumed to play an important role in emotional processing, such as attention to emotion, identification, or regulation of emotion [41], [74]. The altered functional connectivities between visual cortex and prefrontal gyrus have been found in major depression without PD patients, which would pay more attention toward distracting information [71]. The median cingulate gyrus, which are considered to be a key structure of the pain matrix [75], [76], may integrate emotional signal [77]. The region showing a positive activity, when normal adult observed painful stimuli [78]. Consistent with these studies, the abnormal connectivity might indicate that lingual gyrus might partially participate in mood regulation of PDD.

Comparative differences in network connectivity between the PDD group and normal controls may reveal both parkinsonian and depressive characteristics. We found increased brain network connectivity between the prefrontal gyrus and lingual gyrus, decreased connectivity between the limbic system and prefrontal gyrus in the PDD group compared with normal subjects. This difference is consistent with a previous study which showed that the loss of white matter within the cortical–limbic network was positively associated with depression in PD patients [46]. Most studies have shown these regions to be the core mood regulation network regions [44], [57].

Compared with normal subjects, the current study found that the nD-PD patients had decreased functional connectivity between the prefrontal gyrus and the basal ganglia and cerebellum, and increased functional connectivity with the hippocampus and medial cingulate gyrus. Researchers have reported that impaired control could arise from the dysfunction of the basal ganglia, or the thalamus and/or frontal lobes [79]. These results might partially explain the movement disorder symptoms seen in these patients [17].

The limitations of our study include the relatively small sample size and the clinically heterogeneous group. L-DOPA might influence brain activity over time [23], [80], [81]. In our study, all patients with PD were assessed while stopped their medication for 12 hours prior to scanning to minimize the impact of medicine. However, the potentially confounding effects of chronic dopaminergic medications could not be avoided, absolutely elimination of medications influence is impossible. Female sex, younger age of onset and right-sided symptom were previously reported to be risk factors for depression in Parkinson’s disease [82], [83], [84], [85]. PDD and nD-PD group did not differ in age, gender and side of onset, so the impact of those factors did not be considered. All patients had a minor and no patient had a major depressive disorder in this study. Therefore the correlation between the severity of the depression and ReHo with functional connectivity has yet to be revealed in the study. Future studies will need to a large-scale, clinically homogeneity sample to investigate the functional changes of ReHo and connectivity over the evolution of depression in PD.

Taken together, the current results show that patterns of neuronal coherence in resting state were altered in PDD patients. There were widespread differences in the ReHo values between PDD and nD-PD patients within the left middle frontal gyrus, right inferior frontal gyrus, left amygdala and bilateral lingual gyrus. Our findings suggest decreased functional connectivity within the prefrontal-limbic system, and increased functional connectivity in the prefrontal cortex and lingual gyrus in PDD compared with nD-PD group. Therefore, abnormal activity in this region may contribute to the development of depression.
